# Discussion Following the Report of a Case of Trench Feet

**Published:** 1918-02

**Authors:** 


### *Discussion Following the Report of a Case of Trench Feet.*

MM. Quénu, Jacob, Robert, Mauclaire, Routier, Tuffier, Lenormant, Le Fort and Broca. *Bulletins et Mémoires de la Société de Chirurgie de Paris*, Feb. 6, 1917, p. 367.

M. Quénu reported a case in which a wounded colonial developed a case of trench feet with total gangrene of the right foot, as a result of exposure for four days in a shell hole full of water. Additional evidence was thus afforded that humidity played the predominant rôle in causing the condition of trench feet. Q. also remarked that in the cold Vosges region trench feet were exceptional, but that they were common at Verdun, where the trenches were shallow and the men had little opportunity to change their shoes.

M. Jacob recalled the work of Raymond[1] and Parisot[2] to the effect

1. *Archives de Médecine et de Pharmacie Militaires*, Nov. 1915. See p. 255.
2. *Archives de l'Académie des Sciences*, May, 1916. See p. 268.

that trench feet were due to a fungus growth in the water and mud of the trenches and therefore were a form of mycosis, which might be limited and controlled by a quick and appropriate treatment. He agreed that long periods of inactivity in mud and water were the chief cause of trench feet, and he cited the instance of two divisions situated side by side, one on dry and the other on damp ground, all other conditions being the same. There were no cases of trench feet from the dry trenches, but many from the moist ones. He insisted that the mycotic variety of trench feet should not be confused with frozen feet.

M. Robert also remarked that many of the cases he had observed of men coming from the Verdun front, were caused by exposure for several days in shell holes full of mud and water.

M. Mauclaire asked if cases complicated with ascending neuritis had been observed. He stated that it was marked by cutaneous anesthesia which extented high on the limb with very acute pains. A limited hersage of the posterior tibial nerve behind the ankle led to improvement in two cases.

M. Routier asked if a mycosis was thought to be the cause of gangrene, or was it merely contributory. He was inclined to believe it secondary in its effect. He would also like to know whether in men who were not afflicted with trench feet such mycoses were ever present.

M. Tuffier stated that he had examined more than two thousand cases of trench feet. He admitted that the distinction he had previously made between frozen feet and trench feet did not hold, for there often existed a slight degree of actual freezing in the case of men whose feet had been exposed to moist conditions at a temperature considerably above freezing. He believed the condition was due partly to ischemia and partly to infection as stated by Parisot and Raymond.

During the early stages of trench feet, the circulation was in a condition of unstable equilibrium. If at this time proper measures could be taken all serious consequences might be avoided. Prophylactic measures were the best, but unfortunately the frequent relief of the men in the trenches and the proper care of the trenches were not always possible. It was therefore doubly necessary that not only the medical officer, but the other officers as well should be on the lookout for the first symptoms of trench feet. Treatment could then be applied at once. Massage three times a day, cleansing, careful disinfection, and greasing were the essentials. It was of the first importance that treatment should be immediate.

The speaker declared himself convinced that infection played an important part, for he had known of cases in which gas gangrene or severe tetanus developed when there were no lesions except those of the trench foot condition. Such cases, however, were rare in his experience.

M. Lenormant and M. Le Fort stated that they had frequently seen cases in which the condition of trench feet was complicated with neuritis.

M. Quénu insisted upon an essential difference between cases of actual freezing caused by a sudden drop in the temperature and the condition known as trench feet. He said that the latter was produced by complex conditions, and that numbness rather than pain constituted the earliest symptom. He believed that saturation with moisture was the dominant cause, and that a low temperature, compression, and inactivity were favoring conditions. The symptoms might vary according to the importance of one or the other of these conditions.

M. Broca observed that early authorities had differentiated between a condition due to cold and that due to actual freezing. The condition due to cold occurred generally not during the actual freezing temperature but at the time of the thaw, and that this condition, as surgeons attending children often noticed, might result in gangrene.

